# Multisystem inflammatory syndrome in children (MIS-C) and neonates (MIS-N) associated with COVID-19: optimizing definition and management

**DOI:** 10.1038/s41390-022-02263-w

**Published:** 2022-09-01

**Authors:** Eleanor J. Molloy, Natasha Nakra, Chris Gale, Victoria R. Dimitriades, Satyan Lakshminrusimha

**Affiliations:** 1 Discipline of Paediatrics, Trinity College Dublin, the University of Dublin, Trinity Research in Childhood Centre (TRICC) and Trinity Translational Medicine Institute (TTMI), Trinity College Dublin, Dublin, Ireland; 2 Children’s Hospital Ireland (CHI) at Tallaght, Dublin and Neonatology, CHI at Crumlin, Dublin, Ireland; 3 Neonatology, Coombe Women’s and Infants University Hospital, Dublin, Ireland; 4 Division of Infectious Diseases, Department of Pediatrics, UC Davis Children’s Hospital, Sacramento, CA USA; 5 Neonatal Medicine, School of Public Health, Faculty of Medicine, Imperial College London, Chelsea and Westminster Hospital Campus, London, UK; 6 Division of Allergy, Immunology and Rheumatology, Department of Pediatrics, UC Davis Children’s Hospital, Sacramento, CA USA; 7 Division of Neonatology, Department of Pediatrics, UC Davis Children’s Hospital, Sacramento, CA USA

## Abstract

**Abstract:**

During the SARS-CoV-2-associated infection (COVID-19), pandemic initial reports suggested relative sparing of children inversely related to their age. Children and neonates have a decreased incidence of SARS-CoV-2 infection, and if infected they manifested a less severe phenotype, in part due to enhanced innate immune response. However, a multisystem inflammatory syndrome in children (MIS-C) or paediatric inflammatory multisystem syndrome temporally associated with SARS-CoV-2 emerged involving coronary artery aneurysms, cardiac dysfunction, and multiorgan inflammatory manifestations. MIS-C has many similarities to Kawasaki disease and other inflammatory conditions and may fit within a spectrum of inflammatory conditions based on immunological results. More recently neonates born to mothers with SARS-CoV-2 infection during pregnancy demonstrated evidence of a multisystem inflammatory syndrome with raised inflammatory markers and multiorgan, especially cardiac dysfunction that has been described as multisystem inflammatory syndrome in neonates (MIS-N). However, there is a variation in definitions and management algorithms for MIS-C and MIS-N. Further understanding of baseline immunological responses to allow stratification of patient groups and accurate diagnosis will aid prognostication, and inform optimal immunomodulatory therapies.

**Impact:**

- Multisystem inflammatory system in children and neonates (MIS-C and MIS-N) post COVID require an internationally recognized consensus definition and international datasets to improve management and plan future clinical trials.
- This review incorporates the latest review of pathophysiology, clinical information, and management of MIS-C and MIS-N.
- Further understanding of the pathophysiology of MIS-C and MIS-N will allow future targeted therapies to prevent and limit clinical sequelae.

## Introduction

The SARS-CoV-2-associated infection (COVID-19) pandemic, especially secondary to Delta and Omicron variants, has resulted in widespread disease among all age groups including children (<18 years), who account for 16.8% of all cases in the US (CDC COVID data tracker—accessed on January 21, 2022).^1^ SARS-CoV-2 infections appear to have spared children from the most severe illness and deaths with children accounting for 0.156% of nearly 800,000 deaths in the USA.^1^ Typically, with non-COVID infections, children and neonates have the highest risk of sepsis of all age groups, as highlighted in the recent World Health Organization report on sepsis.^2^ Initial concerns that children would be at high risk led to international collaborative consensus efforts on guidelines development, international registries, and clinical trial enrollment,^3^ and advocacy so that children were included in key international SARS-CoV-2 treatment trials such as the RECOVERY trial (The Randomized Evaluation of COVID-19 Therapy).^4^

In newborn infants, fears of severe disease were initially allayed as even mothers positive for SARS-CoV-2 for the most part delivered babies that were usually negative or asymptomatic. Only 1.8–2% of infants born to mothers with COVID-19 test positive in the immediate neonatal period and most of them have minimal signs or complications.^5^ Case reports emerged of severely affected infants, but these were very rare, typically with late-onset neonatal COVID-19.^6,7^

Although COVID-19 was relatively mild in most children, multisystem inflammatory syndrome in children (MIS-C) or pediatric inflammatory multisystem syndrome temporally associated with SARS-CoV-2 (PIMS-TS) subsequently evolved as a post-infectious inflammatory condition associated with abnormal immune function, left ventricular cardiac dysfunction, coronary artery aneurysms, atrioventricular block and clinical deterioration with multiorgan involvement.^8^ The commonly affected age group is children 5–13 years of age, and initially, no cases were described in the early neonatal period.^9,10^ However more recently this syndrome has been increasingly recognized in neonates.^11–15^ In this manuscript, we propose mechanisms and definitions of MIS-C and neonatal multisystem inflammatory disease (MIS-N). We aimed to highlight discrepancies in the definition of MIS-C and paucity of information in MIS-N. We also included discussion of immunological phenotype in MIS-C/MIS-N and the overlap with Kawasaki disease (KD) potentially developing a clinical and immunological phenotype allowing immune targeted therapies.

## Children, COVID-19, and MIS-C: epidemiology^16^

**Fig. 1:**
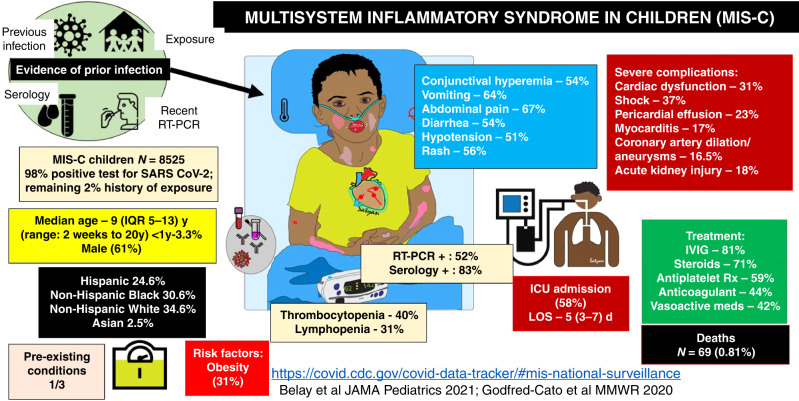
CDC tracker and publications regarding MIS-C in children. Evidence of SARS-CoV-2 infection (current or recent) in the setting of multiple organ system involvement (commonly cardiac, gastrointestinal and mucocutaneous) and elevated inflammatory markers may suggest a diagnosis of MIS-C. Racial and ethnic distribution, risk of ICU admission, management options, and mortality are shown. Data accessed on December 25, 2021. Image courtesy Satyan Lakshminrusimha.

SARS-CoV-2^17,18^ infection in infants and children has been documented in 9,452,491 children in the US (as of January 12, 2022) and most cases are asymptomatic or cause mild clinical illness.^19^ However, at the peak of the pandemic in Europe, UK investigators described a cluster of children with hyperinflammatory syndrome and shock presumed to have developed 2–4 weeks after acute SARS-CoV-2.^20^ On May 14, 2020, Centers for Disease Control and Prevention (CDC) published an online Health Advisory that summarized the manifestations and labeled this condition as an “MIS-C” related to COVID-19. By January 3, 2021, a total of 6431 MIS-C patients had been reported in the US with 55 deaths (Fig. 1).^1^ The majority of these children were Hispanic or black (60%)^10,21^ and presented at a median age of 9 years with abdominal pain, vomiting, diarrhea, skin rash, conjunctival injection, and hypotension.^8,21^ In addition, children with MIS-C had evidence of cardiac dysfunction (40.6%), shock (35.4%), myocarditis (22.8%), coronary artery dilation, or aneurysm (18.6%), and acute kidney injury (18.4%). All patients had RT-PCR or serological evidence of SARS-CoV-2 infection (98%) or contact with someone with COVID-19 (2%).^1,10^ The CDC tracker data on MIS-C (accessed on January 30, 2022), Godfred-Cato et al. and Belay et al. reporting on MIS-C prevalence and characteristics are shown in Fig. 1.^9,10^

The various definitions of MIS-C are shown in Table 1^22,23^ and in general include children who demonstrate persistent fever, involvement of at least two organ systems, laboratory evidence of inflammation, and laboratory confirmation of current or recent SARS-CoV-2 infection. Patients are excluded if they have another plausible explanation for the illness. While the definitions from WHO,^23^ CDC and Royal College of Paediatrics and Child Health in the UK^22^ have considerable overlap, they are not identical and in addition are very similar to KD. An additional proposed definition combining the criteria from these organizations (Table 1) also includes immunization among children resulting in positive serology. Recent evidence also suggests that MIS-C is less common among vaccinated children.^24^

**Table 1:** Criteria for MIS-C diagnosis.

Agency	World Health Organization (WHO)^23^	Centers for Disease Control and Prevention (CDC), USA	Royal College of Paediatrics and Child Health (RCPCH), UK^22^	Proposed uniform definition
Age	0–19 years	<21 years	Child (age not specified)	<21 years
Fever	≥3 days	Fever ≥38.0 °C for ≥24 h, or report of subjective fever lasting ≥24 h	Persistent fever >38.5 °C	Fever ≥38.0 °C for ≥3 days, or report of subjective fever lasting ≥3 days
AND	At least two of the following	≥2 organ system involvement		≥2 organ involvement with specified signs
Clinical features	1 Rash or bilateral non-purulent conjunctivitis or mucocutaneous inflammation signs (oral, hands, or feet) 2 Hypotension or shock 3 Features of myocardial dysfunction, pericarditis, valvulitis, or coronary abnormalities (including ECHO findings or elevated Troponin/NT-proBNP) 4 Evidence of coagulopathy (by PT, PTT, elevated D-dimers) 5 Acute gastrointestinal problems (diarrhea, vomiting, or abdominal pain)	Evidence of clinically severe illness requiring hospitalization, with multisystem (≥2) organ involvement (cardiac, renal, respiratory, hematologic, gastrointestinal, dermatologic, or neurological)	Evidence of single or multiorgan dysfunction (shock, cardiac, respiratory, renal, gastrointestinal, or neurological disorder) Most have oxygen requirement and hypotension Some have abdominal pain, confusion, conjunctivitis, cough, diarrhea, headache, lymphadenopathy, mucus membrane changes, neck swelling, rash, resp symptoms, sore throat, swollen hands and feet, syncope, and vomiting	Cardiac: hypotension or shock, myocardial dysfunction, pericarditis, valvulitis, or coronary abnormalities, pericardial effusion (including ECHO findings or elevated Troponin/NT-proBNP) Gastrointestinal: diarrhea, vomiting, or abdominal pain Mucocutaneous: rash or bilateral non-purulent conjunctivitis, sore throat or mucocutaneous inflammation signs (oral, hands or feet) Hematologic: lymphadenopathy, thrombocytopenia, lymphopenia, evidence of coagulopathy (by PT, PTT, elevated D-dimers) Renal: acute kidney injury Respiratory distress, cough Neurological: confusion, headache, seizures
AND				
Markers of inflammation	Elevated markers of inflammation such as ESR, C-reactive protein, or procalcitonin	One or more of the following: an elevated C-reactive protein (CRP), erythrocyte sedimentation rate (ESR), fibrinogen, procalcitonin, D-dimer, ferritin, lactic acid dehydrogenase (LDH), or interleukin 6 (IL-6), elevated neutrophils, reduced lymphocytes, and low albumin	Inflammation (neutrophilia, elevated CRP, and lymphopenia) Abnormal fibrinogen, elevated CRP, D-dimers or ferritin, hypoalbuminemia, lymphopenia, neutrophilia in most; normal neutrophils in some	One or more of the following (based on age-appropriate cut-offs): an elevated C-reactive protein (CRP), erythrocyte sedimentation rate (ESR), fibrinogen, procalcitonin, D-dimer, ferritin, lactic acid dehydrogenase (LDH), or interleukin 6 (IL-6), elevated neutrophils, reduced lymphocytes, and low albumin
AND				
Absence of other etiology	No other obvious microbial cause of inflammation, including bacterial sepsis, staphylococcal or streptococcal shock syndromes	No alternative plausible diagnoses	Exclusion of any other microbial cause, including bacterial sepsis, staphylococcal or streptococcal shock syndromes, infections associated with myocarditis such as enterovirus	No alternative plausible diagnoses
AND				
Evidence of COVID-19	Evidence of COVID-19 (RT-PCR, antigen test, or serology positive), or likely contact with patients with COVID-19	Positive for current or recent SARS-CoV-2 infection by RT-PCR, serology, or antigen test; or exposure to a suspected or confirmed COVID-19 case within the 4 weeks prior to the onset of symptoms	SARS-CoV-2 PCR testing may be positive or negative	Positive for current or recent SARS-CoV-2 infection by RT-PCR, serology (not explained by prior immunization), or antigen test; or recent exposure to a suspected or confirmed COVID-19 case

Although the median age of MIS-C presentation is 9 years, young infants as early as 2 weeks old presented with MIS-C.^10^ The phenotype of MIS-C appears to vary with the age of the patient with mucocutaneous and gastrointestinal findings more common in younger children and respiratory presentation more common among adolescents. Young children more frequently present with conjunctival findings, rash, and abdominal pain and less commonly with respiratory symptoms. Patient age had no significant effect on the incidence of coronary dilation (18.3% 0–4 years vs. 14.6% at 18–20 years).^25^ In addition, young children with MIS-C had a lower incidence (16–18%) of preceding clinical illness consistent with COVID-19 compared to young adults 18–20 years of age (63%).^25^ Analysis of 85 infants (<12 months, youngest being 2 weeks old) showed that rash (62.4%), diarrhea (55.3%), and vomiting (55.3%) were the most common signs and symptoms.^26^ Serious findings such as hypotension (21.2%), pneumonia (21.2%), and coronary artery dilation or aneurysm (13.9%) led to ICU admission in 32.9% of these infants.^26^ There is considerable overlap between MIS-C and KD with a similar spectrum of disease. The KD phenotype is also reportable to the CDC as often cannot be distinguished from MIS-N/MIS-C and therefore requires discussion in the classification of MIS-C.

## Comparison with Kawasaki disease (KD)

Immediately after cases of MIS-C were reported from Europe in April 2020, many physicians noted similarities between the presentation of MIS-C and KD.^27^ These similarities included symptoms such as fever, mucous membrane changes, rash, conjunctivitis, and lymphadenopathy, as well as frequent cardiac involvement and laboratory tests demonstrating significant inflammation. However, notable differences between these two entities additionally emerged, including disease epidemiology such as the age of affected patients (typically <5 years in KD and 5–13 years in MIS-C).^1^ Patients with MIS-C are more likely to have gastrointestinal manifestations such as abdominal pain, vomiting, and diarrhea,^28^ and the nature of the cardiac involvement is also distinct. In KD, coronary artery aneurysms or dilation and pericardial effusion are most common, whereas depressed ventricular function and highly elevated brain-natriuretic peptide (BNP) are more typical of MIS-C.^29^ Some patients with MIS-C have developed coronary artery dilation and aneurysms, although most of these patients have had complete resolution of the abnormalities at follow-up,^30^ as opposed to KD where a subset of patients have persistent coronary artery abnormalities. Notable differences in laboratory findings include elevated white blood cell count, neutrophilia, and thrombocytosis in KD, as compared to normal white blood cell count, lymphocytopenia, and thrombocytopenia in MIS-C.^8,27,28,31,32^

Given the significant overlap between the 2 entities, and the lack of a definitive diagnostic test for either one, it can be difficult to distinguish between them. Although the presence of a positive test for SARS-CoV-2 is more suggestive of MIS-C, it is clear that SARS-CoV-2 can also trigger KD in some patients.^33^ In addition, a positive SARS-CoV-2 antibody test, which was helpful early in the pandemic to identify patients with prior COVID-19 infection, is more difficult to interpret at this time given widespread infection and vaccination.

## Classification of manifestations of COVID-19 in the neonatal period

**Fig. 2:**
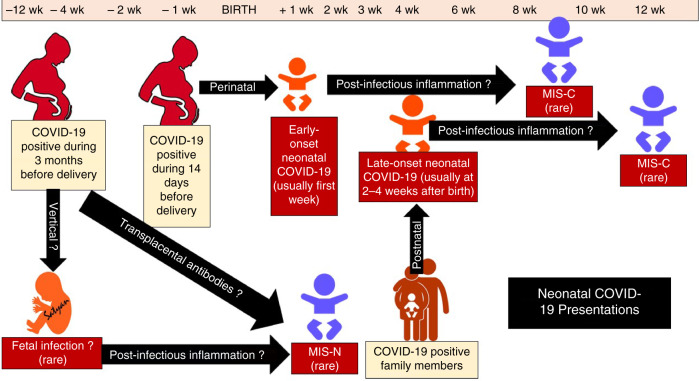
Proposed classification of neonatal COVID-19. The horizontal axis on the top refers to time in relation to birth of the infant. Four presentations of SARS-CoV-2 infection in the neonatal period are shown. Pregnant mother with COVID-19 can rarely result in multisystem inflammation in the neonate either due to fetal infection (speculative) or through transplacental antibodies resulting in multisystem inflammatory syndrome in neonates (MIS-N). Pregnant mothers who are positive for SARS-CoV-2 around the time of birth can transmit infection to the baby in the peripartum period resulting in early-onset COVID-19. Some neonates develop late-onset neonatal infection due to exposure to a family member 2–4 weeks after birth. Infants with early- or late-onset neonatal COVID-19 can potentially develop MIS-C 2–4 weeks later (a rare occurrence).^43^ This classification differentiates MIS-N (secondary to maternal SARS-CoV-2 infection without neonate being RT-PCR or antigen test positive) from MIS-C (secondary to neonatal SARS-CoV-2 infection). Modified from Pawar et al.^11^ Image courtesy Satyan Lakshminrusimha.

Acute SARS-CoV-2 infection and multisystem inflammation have been reported in neonates.^34^ A proposed classification of neonatal presentation of COVID-19 (Fig. 2 and Table 2) and includes early neonatal and late neonatal infection, and MIS-N and MIS-C. We recommend using the term MIS-N to describe neonatal inflammatory illness involving ≥2 organ system involvement and meeting criteria listed in Table 1 along with the maternal history of SARS-CoV-2 infection during pregnancy (Table 2). However, we suggest the exception of fever, which is relatively uncommon in neonates, especially preterm infants where in utero exposure to COVID-19 is the most common source.

**Table 2:** Clinical manifestations of COVID-19 in the neonatal period (with possibility of overlap in some patients).

Condition	Age of neonate at disease manifestation	Source of SARS-CoV-2 infection	Transmission and pathogenesis	Presentation	Diagnosis
Early neonatal COVID-19	Typically, <7 days after birth	Mother	Perinatal leading to acute infection	Respiratory distress, apnea, or asymptomatic^38^	Positive RT-PCR or antigen test from neonate after the first few hours^b^
Late neonatal COVID-19	Typically, 2–3 weeks after birth	Family members (including mother)	Horizontal (postnatal transmission) leading to acute infection	Respiratory distress, congestion, apnea, fever	Positive RT-PCR or antigen test from neonate
MIS-N (?)^a^	Typically, <7 days after birth	Mother (or fetus?)	Transplacental antibodies? Or fetal infection? Leading to an immune-mediated disorder	Multisystem inflammation, coronary dilation,^43^ thrombosis, AV conduction block, ↑ inflammatory markers^11,70^	Meet criteria listed in Table 1 (with the exception of fever) AND evidence of maternal infection with SARS-CoV-2 during the antenatal period
MIS-C	Typically, 2–6 weeks after primary infection	Self (neonate with early neonatal COVID with or without clinical signs)	Primary SARS-CoV-2 infection leads to cytokine or antibody surge leading to an immune-mediated disorder	Multisystem inflammation, coronary dilation, thrombosis, ↑ inflammatory markers	Meet all criteria in Table 1

Modified from Lakshminrusimha et al.^34^

^a^It is not clear if this is truly a distinct presentation of COVID-19 in the neonatal period.

^b^To rule out contamination from maternal secretions.^38,39^

Several investigators have reported different presentations of COVID-19 in the neonatal period. Raschetti et al. presented a systematic review of 176 published cases of neonates suspected to have SARS-CoV-2 infection including 97 neonates with clinical illness.^35^ Among these infants, 44% developed fever and respiratory (53%), gastrointestinal (36%), neurological (19%), and hemodynamic (10%) signs of illness were observed. Most infants had positive RT-PCR for SARS-CoV-2 and two had IgM titers above the threshold.^35^ These cases are consistent with an early-onset acute SARS-CoV-2 infection acquired in the perinatal or early postnatal periods (Fig. 2).^7,36,37^ This presentation is commonly diagnosed with a positive nasopharyngeal swab RT-PCR or elevated IgM titer.^36^ However, nasopharyngeal swabs obtained within 3 h of birth can show false positive results^38^ due to contamination by maternal secretions. For this reason, the American Academy of Pediatrics and Red Book online recommend bathing these newborn babies born to COVID-19-positive mothers soon after delivery to remove the virus potentially present on the skin surface and to test the infant as close to discharge as possible.^39,40^

Late-onset neonatal acute SARS-CoV-2 infection, most likely from postnatal transmission from mother or other family members, has been described.^6^ Fever, poor feeding, and occasionally apnea have been reported with this presentation.^41^

More recently, multisystem inflammation has been described in neonates born to mothers with SARS-CoV-2 infection during pregnancy.^11,13–15,34,42,43^ Given that the typical gap between signs and symptoms of COVID-19 and MIS-C presentation is 27 days (interquartile range, 21–36 days) in older children,^21^ a presentation with multisystem inflammatory syndrome within the first week after birth could be consistent with MIS-N if maternal infection occurred 1–5 weeks prior to delivery and resulted in fetal infection or exposure to antibodies and cytokines (Fig. 2). With MIS-N, most neonates had multisystem involvement, elevated inflammatory markers with positive titers of IgG-SARS-CoV-2. Lack of elevated IgM titers cannot reliably rule out acute infection in neonates, especially preterm infants who may not be able to mount an IgM response due to immunological immaturity.^44^

The diagnostic criteria for MIS during the neonatal period (MIS-N) are controversial and evolving. Lack of clear diagnostic criteria can potentially lead to overtreatment during periods of COVID-19 surge when many pregnant women may test positive for SARS-CoV-2 and have high antibody titers. Our recommended definition is shown in Table 3 and is modified from Pawar et al.^11^ A prior history of infection is also unreliable in neonates because most neonates who test positive for SARS-CoV-2 in the perinatal period have no clinical signs of illness.^38^ Most of the case reports of neonatal MIS-C have relied on a positive IgG titer against SARS-CoV-2 spike protein or confirmed COVID-19 in the mother during the last few weeks of pregnancy. With widespread vaccination, evidence of anti-SARS-CoV-2 antibody may be common secondary to transplacental transfer from immunized mothers or from transfusion of products donated by immunized blood donors. In these cases, detection of antinucleocapsid antibody may be preferred.^15,45^

**Table 3:** Proposed inclusion criteria for neonatal multisystem inflammatory syndrome (MIS-N) secondary to maternal SARS-CoV-2 exposure or infection.

(1) A neonate aged <28 days at the time of presentation
(2) Laboratory or epidemiologic evidence of SARS-CoV-2 infection in the mother
• Positive SARS-CoV-2 testing by RT-PCR, serology (IgG or IgM—and not secondary to immunization), or antigen during pregnancy OR • Symptoms consistent with SARS-CoV-2 infection during pregnancy OR • COVID-19 exposure during pregnancy with a confirmed case of SARS-CoV-2 infection • Serological evidence (positive IgG specific to SARS-CoV-2 but not IgM) in the neonate (and not secondary to maternal immunization)
(3) Clinical criteria
• Meet clinical criteria in Table 1 (except for fever)
(4) Laboratory evidence of inflammation
• Meet inflammatory marker criteria listed in Table 1
(5) No alternative diagnosis (viral or bacterial sepsis; birth asphyxia; maternal lupus etc.) that can explain the clinical features

Modified from Pawar et al.^11^

Infection with SARS-CoV-2 initiates a cell-mediated and humoral immune response that produces antibodies against specific viral antigens such as the nucleocapsid (N) protein and spike (S) protein (such as anti-S protein antibodies that target the spike S1 protein and receptor binding domain-RBD).^46^ IgG and IgM antibodies against S protein can be detected within 1–3 weeks of infection.^47,48^ or vaccination.^46,49^ Detection of anti-S and anti-N antibodies in a previously unvaccinated subject offers reliable evidence of prior infection although 3–4% of infected individuals may not mount an antibody response.^50^ With the CDC recommending the use of SARS-CoV-2 vaccines during pregnancy,^51^ infants born to vaccinated mothers will have elevated IgG titers (maternally derived) against spike protein necessitating better diagnostic tools for the diagnosis of MIS-N in the neonatal period.

**Fig. 3:**
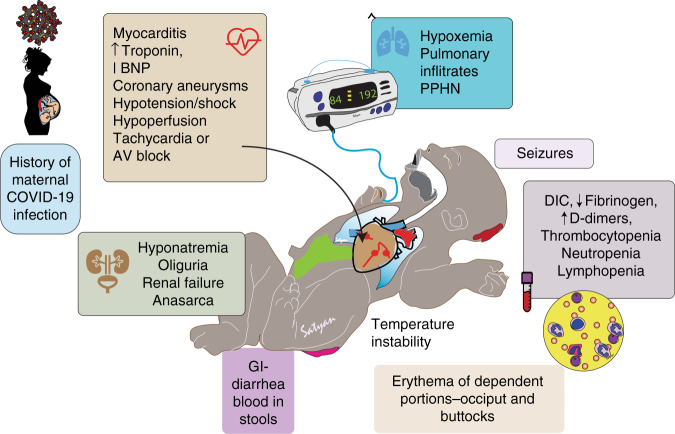
Clinical features of MIS-N or MIS-C in neonates. Maternal infection during pregnancy can be asymptomatic or symptomatic. We speculate that transplacental transfer of antibodies following an autoimmune response in the mother to fetus elicits an autoimmune response in the neonate. This condition is different from early SARS-CoV-2 infection in the neonate. The autoimmune response in the neonate is followed by a multisystem inflammatory response. Typical organ systems involved, and clinical features are shown. Copyright Satyan Lakshminrusimha (adapted from Sankaran et al.^7^). Image courtesy Satyan Lakshminrusimha.

Clinical features (Fig. 3) of MIS-C in neonates and MIS-N range from cardiovascular (myocarditis, coronary arterial dilation, and aneurysms, hypotension, ventricular dysfunction, intracardiac thrombosis), skin rash (vasculitis or due to ischemia), gastrointestinal signs resembling necrotizing enterocolitis (NEC), respiratory distress, persistent pulmonary hypertension of the newborn, neurological signs (hypotonia, lethargy seizures or hypertonia), and conjunctival involvement.^11–15,35,37,42,52,11,53^ These reports suggest that neonatal multisystem inflammatory presentation is more likely to occur in areas where COVID-19 is highly prevalent among pregnant mothers and vaccination rates are low. Pawar et al., More et al., and Shaiba et al. reported five deaths presumably from MIS-N due to cardiac dysfunction/shock, multiorgan failure, or NEC accounting for a mortality of approximately 10% in some case series.^11,15,54^

## Immune responses in MIS-C and MIS-N

Recognition of immune responses is important in potential subclassification of MIS-C to allow targeted treatments and biomarkers. There are significant differences in immune function across age groups that may be associated with the protection from severe acute COVID infection in children and neonates.^3,55,56^ The milder course of the disease in children has been attributed to various factors, including differences in the innate immune system in children, differences in angiotensin-converting enzyme II (ACE2) receptor expression, vitamin D levels,^57^ and previous infections with other common coronaviruses.^55,56^

**Fig. 4:**
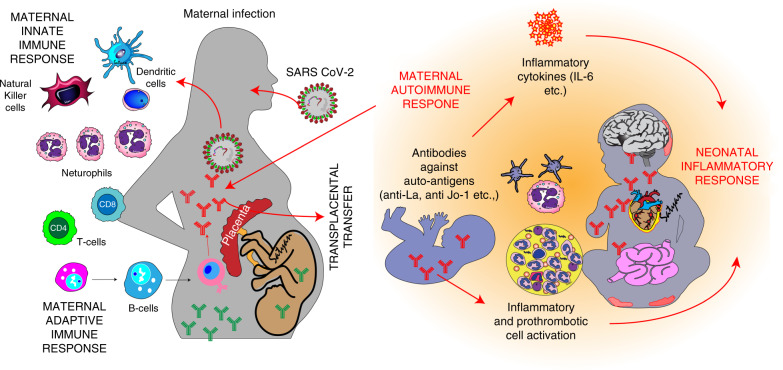
Immunological mechanisms speculated to play a role in MIS-N. In response to a SARS-CoV-2 infection, maternal innate immune system mounts the initial response. Subsequently, maternal adaptive immune response is triggered resulting in formation of antibodies. Antibodies directed against pathogenic areas of the SARS-CoV-2 virus (such as spike protein) are protective (green background). Transplacental transfer of IgG antibodies, particularly those directed toward neonatal autoantigens may be responsible for cytokine release, proinflammatory, and prothrombotic cascade stimulation, and multisystem inflammation (red background). Some neonates may have early acute SARS-CoV-2 infection but may not be able to mount an IgM response due to an immature adaptive immune system. The neonatal autoimmune response triggered against various tissues such as heart, gastrointestinal tract, skin, and mucosa may lead to tissue damage and manifestations of MIS-N. Image courtesy Satyan Lakshminrusimha.

The host response to SARS-CoV-2 infection involves both innate and adaptive immunity, with early response relying on innate activation to initiate further T and B cell responses (Fig. 4). Many aspects of innate immunity, including the role of ACE2 expression and viral infections in children, are relevant in modulating the antibody response to SARS-CoV-2 infection. Ultimately, the development of neutralizing antibodies in conjunction with adaptive cell memory response to SARS-CoV-2 is associated with some amount of protection.^58^ Goenka et al. recently reported distinct immune responses in infants <12 weeks old with fever compared to their parents and adults who had recovered from confirmed SARS-CoV-2 infection. Infants had robust functional antibody response but, as would be expected in the immature immune system of the neonate, their peripheral blood mononuclear cell IFN-g responses were decreased; this factor could ultimately have protected these infants from severe COVID-19.^59^

However, the T cell subsets and inflammatory profile seen in children with MIS-C differs from what is observed in children with acute SARS-CoV-2 infection. Furthermore, increases in specific chemokines and cytokines help to distinguish MIS-C from active SARS-CoV-2 infection or KD.^60^ In this study by Consiglio et al., children with SARS-CoV-2 infection (*n* = 41) or MIS-C (*n* = 13) were compared to healthy controls (*n* = 19) and children with KD (*n* = 28) using systems-level analysis of cytokines, autoantibodies, and peripheral immune cells. Children with MIS-C were significantly older (5–15 years), had higher CRP and ferritin, and lower platelet and leukocyte counts compared to the other groups (and in accordance with previous clinical reports). Of interest, although those patients showed adequate serological responses to SARS-CoV-2, pre-existing immunity to other coronaviruses was lower. There was some overlap in the hyperinflammatory responses of children with MIS-C and KD, although they had lower IL-7 and IL-8 compared with ICU-admitted adults with acute SARS-CoV-2 infection. IL-17a was an important mediator in the cytokine storm in KD but not the inflammatory response of MIS-C, where cytokines such as tumor necrosis factor beta (TNF-b), ITGA11, and CCL25 responded best to immunomodulation. In other studies, however, pediatric patients with MIS-C were found to have elevations in both IL-17a as well as IL-6 cytokines in their immune signatures.^61^

Mapping of the anti-SARS-CoV-2 antibody response in MIS-C is similar to a convalescent response, with evidence of myeloid chemotaxis and mucosal immunity.^61^ Notably, MIS-C patients were reported to have significantly higher titers of IgG receptor RBD antibodies to SARS-CoV-2 spike protein compared to patients with acute COVID-19, KD, or healthy controls.^62^ In contrast, spike protein RBD IgM antibody titers were similar across all the patient profiles.^62^ This may suggest an important role for IgG antibodies in MIS-C as some of these antibodies might be involved in triggering a subsequent immune response.

In considering possible pathologic consequences of antibody responses, patients with MIS-C have high levels of certain antibodies against autoantigens (anti-SSB, anti-Jo-1), lending credence to the hypothesis that MIS-C is mediated by a persistent autoimmune response to the original infection.^61^ As such, and analogous to neonatal lupus, where anti-SSA and anti-SSB antibodies cross the placenta to cause manifestations such as rash and congenital heart block in newborns, it is a plausible theory that similar autoantibodies against tissue-based antigens could cross the placenta after a SARS-CoV-2 infection and initiate MIS-N disease in a neonate. These antibodies should be distinguished from protective IgG antibodies against spike protein of the virus that is also transmitted across the placenta (and IgA in breastmilk) in response to infection and vaccination in pregnant mothers (Fig. 3).^63^

Currently published data on immune function does not include infants <11 months. There is a paucity of data on newborn immune responses and especially MIS-N.^16^ Further exploration of detailed immune function and cell phenotype is required to establish a better understanding and to develop appropriate immunotherapies for children and neonates with the multiorgan inflammatory syndrome.

## Management of MIS-C in children and neonates

Due to a lack of prospective data to inform the treatment approach to MIS-C and MIS-N, North American treatment protocols have largely been based on the treatment of KD and other inflammatory/autoimmune disorders.^64^ Most centers have chosen either glucocorticoids, intravenous immune globulin (IVIG), or a combination of those drugs for initial treatment, while reserving biologic agents such as IL-1 receptor antagonists (i.e., anakinra), TNF-alpha blockers (i.e., infliximab), or IL-6 receptor antagonists (i.e., tocilizumab) for refractory cases.

The American College of Rheumatology has provided guidance regarding treatment, and note that a “stepwise” approach is recommended, and some patients with mild symptoms may not require any treatment but only close monitoring. First-line treatments for hospitalized patients include IVIG and low-moderate dose corticosteroids^65^ updated guidelines February 3, 2022 available at: https://www.rheumatology.org/Portals/0/Files/ACR-COVID-19-Clinical-Guidance-Summary-MIS-C-Hyperinflammation.pdf. In our opinion, steroids are often adequate for mild disease, with the addition of IVIG for patients with moderate-severe disease such as cardiac ventricular dysfunction, or for patients meeting diagnostic criteria for KD or with coronary artery dilation or aneurysms (Table 4). Of note, in an international observational cohort study of 614 patients, McArdle et al. found that there were no significant differences in outcomes between patients who were treated with IVIG, IVIG plus glucocorticoids, or glucocorticoids alone.^66^ The RECOVERY trial has included children >44 weeks postmenstrual age with PIMS-TS in a randomized comparison between IVIG and high-dose methylprednisolone, with results expected imminently. IVIG in MIS-C is thought to reduce autoantibody-mediated pathology by activating inhibitory Fc-receptors stopping complement causing membrane attack complexes.^67,68^ High-dose (2 g/kg) IVIG is recommended, similar to KD, although the dose may need to be divided in the setting of significant cardiac dysfunction.

**Table 4:** Therapeutic options for MIS-C in children.^65^^,^^71^

	Mild disease	Moderate-severe disease^a^
Steroids	Methylprednisolone 2 mg/kg/day IV divided q12h (max 60 mg/day) for the first 5 days, then transitioned to oral prednisone and tapered over 2 weeks	Methylprednisolone 10–20 mg/kg/day IV divided q12h on the first day (max 500 mg/day), followed by 2 mg/kg/day IV divided q12h (max 60 mg/day) for days 2–5, then transitioned to oral prednisone and tapered over 3–6 weeks^b^
IVIG	Only if patient meets the criteria for Kawasaki disease (including incomplete definition as per AHA^72^) or has coronary artery dilation or aneurysm	Yes: 2 g/kg—based on ideal body weight—can be divided into two doses if concerns about LV dysfunction^c^
Anakinra	No	For severe or refractory cases consider 2–10 mg/kg/day IV or SQ for a minimum of 5 days (or longer depending on the clinical response)^d^
Anti-platelet therapy and anticoagulation	Low-dose aspirin	Prophylactic enoxaparin. Aspirin may be added per cardiology discretion
GI protection (i.e., H2 blocker)	Yes	Yes

^a^Moderate-severe disease defined as: need for vasoactive medications or inotropes, mechanical ventilation, significantly decreased LV function, ICU admission.

^b^RECOVERY trial (recoverytrial.net)^73^ used methylprednisolone sodium succinate 10 mg/kg (as base; maximum, 1 g) once daily for 3 days.

^c^RECOVERY trial (recoverytrial.net)^73^ used 2 g/kg as a single dose (based on ideal body weight in line with NHS England guidance) for children with corrected gestational age >44 weeks and <18 years with PIMS-TS phenotype.

^d^RECOVERY trial (recoverytrial.net)^73^ used 2 mg/kg daily for 7 days or until discharge whichever is sooner for children with PIMS-TS (>12 months of age and >10 kg body weight).

Serial laboratory monitoring every 24–48 h is recommended to assess response to treatment, including C-reactive protein, complete blood count, D-dimer, ferritin, troponin T, and BNP. Tapering of immunomodulatory therapy is recommended, typically over a 2–3-week period, and should be guided by clinical, laboratory, and echocardiographic response. Low-dose aspirin is recommended for all patients, although patients with moderate-severe disease or other risk factors for thrombosis should receive prophylactic anticoagulation (Table 5).^67^ For patients with ongoing refractory shock, fever, or inflammation despite treatment with IVIG and steroids, the addition of anakinra or higher doses of glucocorticoids is recommended. Depending on clinical status, electrocardiogram (EKG) and/or echocardiography may be repeated while the patient is still hospitalized. Discharge is recommended once a patient has been afebrile for 48 h with improving inflammatory markers and resolving multisystem organ involvement.

**Table 5:** Recommendations for anti-platelet therapy and anticoagulation.^67^

Mild disease	All patients with MIS-C should receive low-dose aspirin. Patients who have mild disease do not need thromboprophylaxis with enoxaparin^a^ unless: (1) D-dimer ≥5 times the upper limit of normal OR (2) additional venous thromboembolism (VTE) risk factors: age ≥12 years, obesity, complete immobilization, central line, estrogen therapy, family history of VTE
Moderate-severe disease	Recommend prophylactic^b^ management with enoxaparin or unfractionated heparin (UH)^c^ unless otherwise contraindicated (platelet count <50,000, fibrinogen <100 mg/dL, major bleeding) • Once patient is clinically stable (generally when they are transferred to general pediatric ward), they can be changed to aspirin unless they meet any of the criteria listed above
Hematology consult	• Rapidly increasing D-dimers • Prior history of VTE • Patients with significant underlying medical conditions (i.e., malignancy, sickle cell disease or other hemoglobinopathy, cardiac disease, nephrotic syndrome, CF, autoimmune disease) • Patients with suspected or confirmed VTE or pulmonary embolus
Discharge recommendations	• Consider stopping anticoagulation with enoxaparin at discharge unless patient has known VTE, central line, D-dimer remains ≥5 times the upper limit of normal, or other medical conditions. All patients should continue low-dose aspirin until cardiology follow-up

^a^For patients who do not meet requirements or are contraindicated for use with enoxaparin or UH, consider early ambulation and/or the use of sequential compression devices (SCDs).

^b^If patients were previously on prophylactic dosing of enoxaparin or UH, they should be increased to treatment dosing.

^c^For initiation of heparin, consult hematology and pharmacy to dose.

In descriptive reports, management of MIS-C has included use of IVIG (80.5%), steroids (62.8%), anti-platelet medications (58.6%), and anticoagulants (44.2%).^9,10,25^ Approximately two-fifths of these patients received treatment with vasoactive medications.^10^

Two to four weeks after discharge, follow-up with pediatric cardiology is recommended for repeat cardiac studies including EKG and echocardiography. A follow-up appointment with either pediatric infectious diseases or rheumatology is typically scheduled close to the completion of a patient’s steroid course or other immunomodulatory therapy. Follow-up with pediatric hematology is recommended for patients who are discharged on enoxaparin to determine the duration of anticoagulation.

Neonatal management of MIS-C/MIS-N is predominantly supportive. The therapies listed above for older children have been used in neonates, and treatment should be focused on critically ill neonates—especially those presenting with shock, myocardial and coronary involvement. Caution should be exercised during IVIG treatment in neonates due to the potential risk of NEC.^69^ More common causes for cardiac dysfunction and elevated Troponin or BNP such as perinatal asphyxia and bacterial infection should be considered. The use of glucocorticoids and IVIG in neonates with MIS-C is not based on current evidence and warrants further study, in particular given the link between high-dose corticosteroids and neurodevelopmental impairment seen in preterm infants when given to prevent bronchopulmonary dysplasia; neonates <44 postmenstrual weeks were not included in the RECOVERY trial comparing IVIG and high-dose methylprednisolone for PIMS-TS for this reason.

## Future directions

Further collaborative networks for children with MIS-C or MIS-N to understand the immune function and optimal therapies are vital.^70,71^ Although there has been excellent progress on maternal and neonatal registries, MIS-C and MIS-N do not yet have an internationally used definition or management plan.^4,72–74^ Understanding immune function would also allow the monitoring of disease progress and response to therapy with biomarkers such as cytokines. This review has highlighted variations in the definition of MIS-C and also the lack of data on a definition of cases with MIS-N. Therefore, a consensus on these definitions internationally would allow optimal international comparisons and lead to strategies for collaborative research in therapies. The next stage could be a Delphi online questionnaire with a broad sample of healthcare professionals and families followed by consensus meetings to clarify the definition.

## Conclusion

Multisystem inflammation following COVID-19 is not common but can be associated with high morbidity and mortality. Neonatal presentation of MIS-C can be either due to early-onset COVID-19 in the neonate or maternal infection (MIS-N). Diagnostic criteria and treatment of neonatal multisystem inflammation secondary to COVID-19 proposed in this article are likely to evolve and readers are recommended to review updated literature.
